# Supplementary material for: Vitamin D Modified DSS-Induced Colitis in Mice via STING Signaling Pathway
Source: Biology (Basel). 2025 Jun 18;14(6):715. doi: 10.3390/biology14060715 (PMC12190092; doi:10.3390/biology14060715)
Supplement: Supplementary file 1 [file biology-14-00715-s001.zip › Supplementary Materials S1: 16s-report of gut microbiota/Groups/diff_analysis/metagenomeRMETACYC/figure/barplot/top20.pdf]

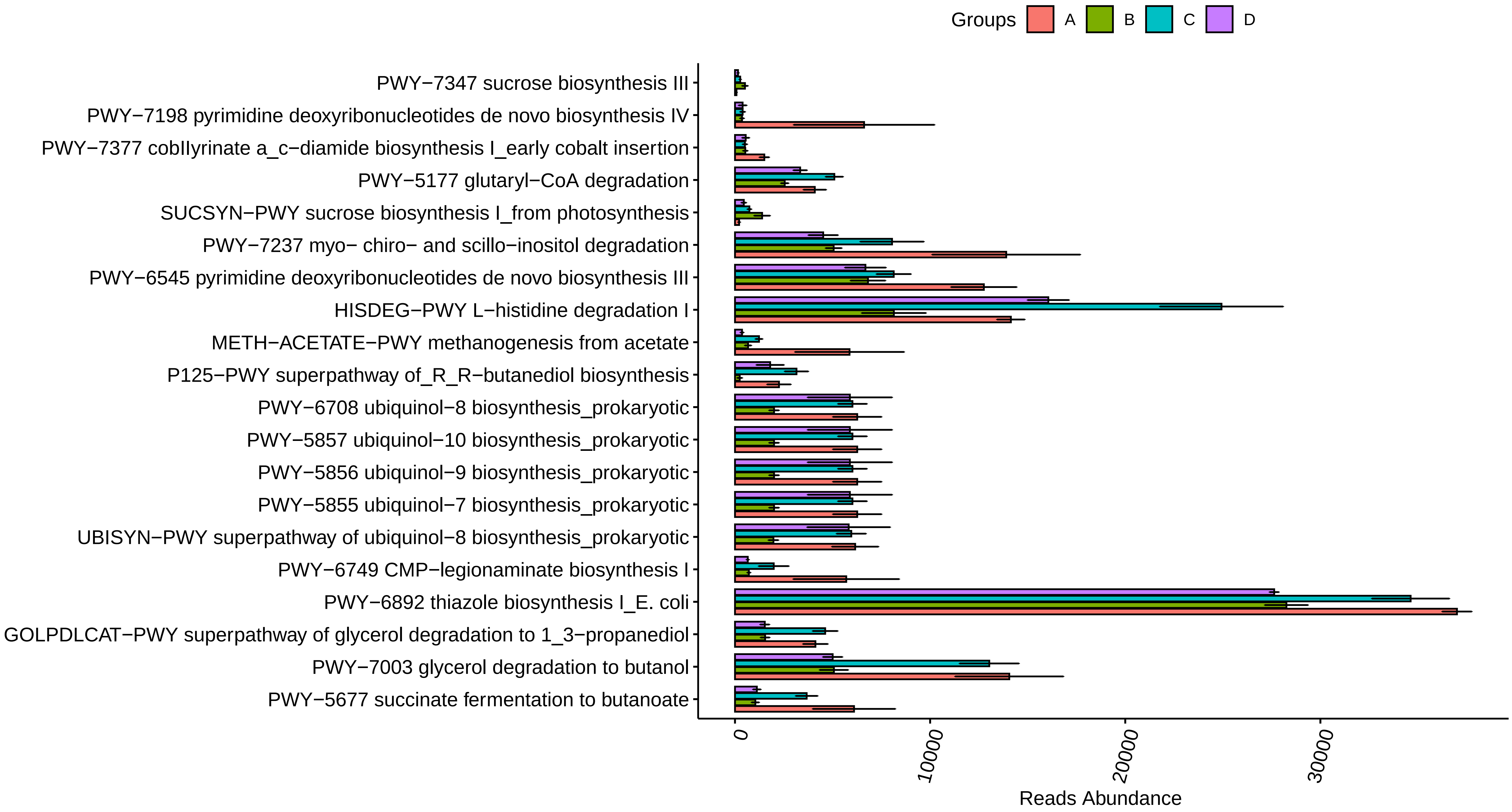
Groups
A
B
C
D
PWY−7347 sucrose biosynthesis III
PWY−7198 pyrimidine deoxyribonucleotides de novo biosynthesis IV
PWY−7377 cobIlyrinate a_c−diamide biosynthesis I_early cobalt insertion
PWY−5177 glutaryl−CoA degradation
SUCSYN−PWY sucrose biosynthesis I_from photosynthesis
PWY−7237 myo− chiro− and scillo−inositol degradation
PWY−6545 pyrimidine deoxyribonucleotides de novo biosynthesis III
HISDEG−PWY L−histidine degradation I
METH−ACETATE−PWY methanogenesis from acetate
P125−PWY superpathway of_R_R−butanediol biosynthesis
PWY−6708 ubiquinol−8 biosynthesis_prokaryotic
PWY−5857 ubiquinol−10 biosynthesis_prokaryotic
PWY−5856 ubiquinol−9 biosynthesis_prokaryotic
PWY−5855 ubiquinol−7 biosynthesis_prokaryotic
UBISYN−PWY superpathway of ubiquinol−8 biosynthesis_prokaryotic
PWY−6749 CMP−legionaminate biosynthesis I
PWY−6892 thiazole biosynthesis I_E. coli
GOLPDLCAT−PWY superpathway of glycerol degradation to 1_3−propanediol
PWY−7003 glycerol degradation to butanol
PWY−5677 succinate fermentation to butanoate
0
10000
20000
30000
Reads Abundance
